# Heart Disease

**Published:** 1890-10-25

**Authors:** 


### The Practitioner's Mirror and Retrospect.

[*The Editor will be glad to receive offers of co-operation and contributions from members of the profession. There is no doubt that much valuable material full of interest and instruction is at present lost to science. This is largely so in the case of* (1) *the younger and less-known members of the hospital staff* (2) *those connected with cottage hospitals, and* (3) *the great body of medical practitioners not attached to any institution. The co-operation of all is cordially invited to enable the Editor to make* The Hospital *of the utmost possible use and value to the profession. Specialists willing to review books on their special subjects are invited to communicate this fact at once. All letters should be addressed to* The Editor, The Lodge, Porchester Square, London, W.]

### THE LONDON POST-GRADUATE COURSE AT THE PADDINGTON INFIRMARY.

### HEART DISEASE.

Dr. Bristowe gave the second lecture to the London Post-Graduate Course on Monday afternoon. After thanking the medical superintendent of the infirmary (Dr. Savill) for giving him the opportunity of lecturing at the Paddington Infirmary, he proceeded to the subject of his lecture, which he entitled "Groups of Cardiac Diseases." He especially entered into the significance of three murmurs, namely, *a*, diastolic mitral; *b*, diastolic aortic; and *c*, haemic, or murmurs which were present without any organic lesion of the heart. Commencing with the diastolic mitral, Dr. Bristowe referred to the paper that he read before the Medical Society a few months ago, in which he distinguished three varieties of this murmur—1, produced during the early part of the diastole, immediately following the second sound of the heart; 2, those murmurs occurring during the intermediate period; and, 3, those which ran up to the first sound; that is to say, the true diastolic, the intermediate, and the true presystolic murmurs. A consideration of these led up to another point, namely, when all these murmurs coincided and blended in the same subject, and so, instead of short ones, as above described, one murmur occupying the whole of the diastolic period was present; in this way there might be considered to be four varieties.

(*a*) He then first considered the true presystolic, which was produced by the systole of the auricle immediately preceding the systole of the ventricle, and was in fact an auriculo-systolic murmur. In this presystolic murmur there was a tendency to the production of a murmur during the whole period, but it was especially liable to occur towards the end. He said that for the sake of convenience one could speak of an early diastolic mitral, a mid-diastolic mitral, and a late diastolic mitral murmur. With a contracted mitral valve as revealed by this presystolic murmur the contraction of the two sides of the heart as a rule was not quite synchronous and so a reduplication of the second sound was produced, and when this happened the second element in the reduplication indicated the contraction of the aortic valve. He had two examples of these lesions to show, one from the infirmary and another he had brought with him from St. Thomas's Hospital. The infirmary patient was a woman in No. 5 ward, aged 29. There was a presystolic murmur present a little above the apex; at the apex itself the murmur occupied a longer period, beginning with the second sound, increasing in intensity sometimes ran up to the first sound, and at other times became attenuated and ended with the first sound. In the second case, that of a small boy he had brought with him, there was a true presystolic murmur at the apex, and between the nipple and the sternum a galloping sound, that is, a sound following the first sound and succeeded by another. The first sound itself was free from murmur, the second sound or the first element of this was also free from murmur, then came the reduplication of the second sound, following a slight murmur. He said that the second sound at the aortic orifice was often lost in these cases midway between the apex and the base.

(*b*) Diastolic Aortic Murmurs.—There were two cases illustrating this condition in the infirmary. This murmur begins with the second sound and dies away very gradually; it is usually best heard to the left of the sternum, or mid-way between the nipple and the sternum, and occasionally at the apex, and in these cases it may be mistaken for a mitral systolic. The reason why it is not so well heard at the aortic orifice itself, as is the case with the systolic murmur of the same region, is because the murmur is carried down the with the regurgitating stream of blood. With aortic regurgitation there was the well-known collapsing pulse, the pulsation of the arteries in the neck, and the presence of the capillary pulsation. The last was usually shown by rubbing the forehead, but it was more easily seen by pressing a glass slide on to the lip, cheek, or ear, and so pressing some of the blood from the region, and then the alternate filling and emptying of the fine capillaries was very readily demonstrated. The first case of this disease he had to show was that of a girl, R—, aged 15. She had had rheumatism ten months ago, and her illness dated from that attack, she had a mitral systolic murmur and also a slight thrill at the apex, and so possibly also some obstructive mitral disease as well as a regurgitant. There was also a diastolic murmur, but not very loud, and this was loudest at the apex, together with a water-hammer pulse, and marked capillary pulsation, and in this case the second sound at the base could be heard over a considerable area. Broadbent has stated that the actual loudness of the sound in aortic regurgitation was no proof of the amount of regurgitation; here there was a loud second sound and abundant regurgitation. The second case illustrating aortic disease was that of a man G——. He had never had rheumatism, and had a slight aortic systolic murmur and a marked diastolic one, which was loudest towards the lower part of the sternum. The patient had also a collapsing pulse and pulsation of the vessels in the neck, but no marked capillary pulsation. Dr. Bristowe said the reason why in one case an aortic diastolic murmur was heard in one spot, and in another case in another, was because of the different direction of the blood current of regurgitation, and this was due to the modification given to the stream by the condition of the valves. The pulsation of the vessels in the neck was not diagnostic of aortic disease as it sometimes occurred after any of the acute fevers, and also in anæmia.

(*c*) Hæmic murmurs. The case of K. A. was then described. She had had rheumatism five years previously, since this she had been ill with dyspnœa. She had a systolic apical murmur, possibly a systolic at the base, but this, if present was so slight that one could not be certain about it; there was also a murmur at the left of the sternum, and this was probably a hæmic murmur, prediastolic in time. These hæmic murmurs were often very puzzling, and Dr. Bristowe referred to a case in St. Thomas's some years ago, which presented a loud rasping murmur at the apex and towards the bases this diminished and almost disappeared, over the aortic area there was a sudden increase in intensity of the murmur. The conclusion was, that the almost complete disappearance of the murmur between the apex and base pointed to there being two separate lesions, one mitral and the other aortic, notwithstanding that the sounds at both orifices were of the same intensity. The patient died, and it was then found that there was rupture of one of the chordae tendineae, that the aortic valves were healthy, there being a lesion only at the mitral orifice, and hence the murmur heard at the aorta was only the conducted mitral one. This third group of murmurs, namely, hæmic, were sometimes said to be pericardial in origin, but there was but rarely any pericardial mischief to be found in these cases. What their exact meaning was, as yet, it was impossible to say.

After the lecture Dr. Bristowe went into the wards, and personally demonstrated the cases he had described in the lecture.

